# The Mechanism and Clinical Application of Constraint-Induced Movement Therapy in Stroke Rehabilitation

**DOI:** 10.3389/fnbeh.2022.828599

**Published:** 2022-06-21

**Authors:** Dong Wang, Junlu Xiang, Ying He, Min Yuan, Li Dong, Zhenli Ye, Wei Mao

**Affiliations:** ^1^Affiliated Hospital of Chengdu University, Chengdu, China; ^2^Chengdu Women’s and Children’s Central Hospital, Chengdu, China; ^3^Chengdu Integrated TCM and Western Medical Hospital, Chengdu, China

**Keywords:** CIMT, stroke, rehabilitation, mechanism, clinical application

## Abstract

Constraint-induced movement therapy (CIMT) has been widely applied in stroke rehabilitation, and most relevant studies have shown that CIMT helps improve patients’ motor function. In practice, however, principal issues include inconsistent immobilization durations and methods, while incidental issues include a narrow application scope and an emotional impact. Although many studies have explored the possible internal mechanisms of CIMT, a mainstream understanding has not been established.

## Introduction

Stroke is one of the main causes of disability in China. Most stroke patients have long-term and significant limb dysfunction that limits their activities and social participation (Wang et al., 2019). Therefore, the recovery of limb motor function is the main goal of stroke rehabilitation, and thus, it is very important to explore effective strategies that can facilitate the rehabilitation of limb motor function after a stroke (Zhang et al., 2020).

Constraint-induced movement therapy (CIMT) is a neurological rehabilitation treatment designed to improve upper extremity motor functions after stroke. The basis of CIMT is improving the function of the affected limb after a stroke by restricting the use of the healthy limb and forcing the use of the affected side. The core strategy of CIMT is the application of movement techniques, behavioral techniques and restriction methods to increase the frequency of use of the affected limb in stroke patients, improve the quality of movement of the affected limb in real-life scenarios, prevent or correct the learned non-use of the affected limb, and promote the recovery of motor function in the affect limb. This method induces the use of the affected limb, corrects or reverses habitual disuse and neglect of the affected limb, and provides structural and functional training and repeated practice opportunities for the affected limb. As a rehabilitation method involving repetitive intensive unilateral limb training, the efficacy of CIMT in improving the motor function of the affected limb has been confirmed by many studies (Hu and Bai, 2020). Taub and coworkers have provided considerable contributions to systematic research on the clinical application and mechanisms of CIMT and have promoted its development and implementation. Their technological contribution is the transfer package (TP) technique, which refers to the transfer of therapeutic gains from CIMT from the controlled treatment setting to daily life. Their contributions to elucidating CIMT mechanisms include the findings that CIMT can improve white matter integrity (Barghi et al., 2018) and increase gray matter in motor areas of the brain (Gauthier et al., 2008).

However, it has been gradually revealed through long-term clinical practice and application that CIMT may have potential shortcomings in terms of comfort, convenience, aesthetics, and fall risk (Daniel et al., 2012). Basic research has also found that differences in the types of restrictions, duration of restriction, intervention time, training intensity, and evaluation methods weaken the evidence for the clinical value of CIMT; therefore, it is difficult to draw high-quality conclusions regarding treatment efficacy and CIMT intensity (Sirtori et al., 2009; Kwakkel et al., 2015). Some scholars have also noted that CIMT may overemphasize the individual movement of the affected upper limb and ignore the cooperative movement of both upper limbs. Although CIMT can effectively improve the function of the affected limb, the therapeutic effect does not necessarily transfer to daily life (Nijland et al., 2011; Taub and Uswatte, 2014).

Therefore, the application of CIMT after a stroke remains controversial. In this paper, the application of CIMT in clinical and basic research is comprehensively reviewed to provide a reference for clinical and basic research on poststroke CIMT.

## The Mechanisms of CIMT in Poststroke Recovery

Results from clinical practice have indicated that CIMT can improve the limb function of stroke patients, and many basic studies have investigated the mechanisms of CIMT from the aspects of neurological function and angiogenesis. Methods such as electrophysiology and imaging were also used.

### Study on the Internal Mechanisms of CIMT

#### Improvements in Neurological Function

Improving neurological function has been the main focus of recent research on CIMT in improving poststroke function. Hu et al. (2021) found that CIMT can promote the recovery of motor function in the injured upper extremity after stroke by enhancing AMPAR-mediated synaptic transmission in the ischemic hemisphere. The same team also found that CIMT can enhance the plasticity of dendrites and dendritic spines in the ipsilateral and contralateral sensorimotor cortex and increase the expression of GluR2 in the ipsilateral sensorimotor cortex synapse (Hu et al., 2020). Liu et al. used a pseudorabies virus to label the efferent neural network that innervates paralyzed forelimbs and explored the recruitment of neurons. Their results showed that CIMT significantly improved the ability of rats to walk proficiently and induced more neurons to re-enter the neural network of the contralateral paralyzed forelimb. CIMT also increased the number of synapses in the contralateral cortex but did not do so in the intact ipsilateral cortex (Liu et al., 2019). Nesin et al. used Golgi-Cox staining to show a significant reduction in dendritic arborization in the damaged motor cortex after stroke. CIMT effectively reversed this effect, and the increase in dendrites was more notable in L3 pyramidal neurons. Biotinylated dextran amine tracking showed that CIMT promoted the reconstruction of interhemispheric axonal connections (Nesin et al., 2019). Zhao et al. (2013) observed that CIMT did not reduce the infarct volume but significantly increased the length and number of midline crossings between contralateral corticospinal axons and the denervated cervical spinal cord. Livingston-Thomas et al. (2014) found that the proportion of BDNF-expressing cells in the peri-infarction area did not change after CIMT intervention but that the source of BDNF-expressing cells changed, resulting in a significant increase in the non-neuronal and non-astrocytic expression of BDNF; the speculated origin is microglia. The close relationship between cortical reconstruction and CIMT has also been reported (Taub et al., 2014).

Therefore, the known mechanisms of CIMT in the poststroke reconstruction of neurological function mainly include an increase in the number of synapses, an increase in dendritic arborization in the motor cortex, and changes in neurotrophic factors.

#### Angiogenesis

Poststroke angiogenesis is an important step in functional reconstruction. Li et al. (2017) found that CIMT promoted neurogenesis and angiogenesis by increasing the expression of endogenous hypoxia-inducible factor-1α and vascular endothelial growth factor and ultimately induced neuroprotection and functional recovery after cerebral ischemia. Zhai and Feng (2019) have demonstrated that compared with fasudil, CIMT resulted in better angiogenesis, nerve regeneration and nerve function recovery at 4 weeks after cerebral ischemia/reperfusion. These results indicate that angiogenesis plays an important role in the promotion of poststroke rehabilitation by CIMT.

#### Other Internal Mechanisms

Gao et al. found that modified CIMT (mCIMT) upregulated the expression of glutamate ionotropic receptor AMPA type subunit 3 in the hippocampus and downregulated the expression of the β3 adrenergic receptor gene Adrb3 and arginine vasopressin receptor 1A. In addition, they also found that mCIMT effectively reduced the glutamate content in the contralateral hippocampus (Gao et al., 2020). In addition, Zhang et al. (2015) found that CIMT significantly improved the function of the forelimbs in rats, an improvement that may be related to the reduction in the expression of phosphorylated extracellular signal-regulated kinases in the bilateral cortex and hippocampus.

### Electrophysiology and Imaging

The use of electrophysiological and imaging methods has more intuitively demonstrated the effect of CIMT after a stroke. Joo et al. used motor evoked potentials (MEPs) and somatosensory evoked potentials (SEPs) to evaluate CIMT-induced electrophysiological changes in infarcted rats. The SEP waveform in rats in the cerebral infarction group was reversed and delayed, and the SEP waveform in rats in the CIMT group was retained, with only a decrease in amplitude. The results showed that CIMT promoted the recovery of motor function in patients after focal cerebral infarction, an effect that may be related to the reorganization of the brain neural network in the somatosensory pathway (Joo et al., 2012). In addition to MEPs, diffusion tensor imaging has also been applied to study the effect of CIMT on corticospinal tract (CST) fibers in ischemic rats. Hu et al. (2019) used diffusion tensor imaging to quantify the fractional anisotropy and mean diffusion coefficient of the CST before and after CIMT in stroke rats and found that CIMT promoted functional recovery after an ischemic stroke by promoting the reconstruction of the ipsilateral CST. Using micro-positron emission tomography/computed tomography imaging, Li et al. found that CIMT improved the behavioral results of cerebral ischemic rats, an improvement that may be related to an increase in glucose utilization in the contralateral cerebral hemisphere (Li et al., 2018). Focal transcranial magnetic stimulation has been used to map the cortical motor output area of a hand muscle on both sides in 13 stroke patients in the chronic stage of their illness, serving as the first human demonstration of a long-term alteration in brain function associated with a therapy-induced improvement in movement rehabilitation after neurological injury (Liepert et al., 2000). Electroencephalography and electromyography monitoring results have shown that the effect of CIMT on poststroke patients were closely related to changes in movement-related cortical potentials (Miltner et al., 2016). Gauthier et al. (2008) found that CIMT significantly increased the gray matter in the ipsilateral and contralateral sensory and motor domains through MRI and that the extent of the increase in gray matter was positively correlated with the degree of improvement in arm function. Barghi et al. (2018) found that CIMT improved white matter integrity through MRI and tract-based spatial statistics (TBSS) in continued research on structural white matter changes.

## Clinical Application of CIMT in Poststroke Rehabilitation

### Application of CIMT for the Upper Limbs

In clinical practice, CIMT is mainly used to improve poststroke upper limb dysfunction by promoting the use of functionally impaired limbs after a stroke. Some scholars compared the efficacy of a low-intensity mCIMT regimen and that of a conventional rehabilitation regimen in the treatment of paresis in stroke patients. The results showed that the overall improvements in Wolf Motor Function Test–functional ability, Motor Activity Log–amount of use and Motor Activity Log–quality of movement were higher in the mCIMT group than in the control group. The difference between the two groups was significant after treatment and at the 3-month follow-up (Smania et al., 2012). In the long-term observation of the role of CIMT, Wolf et al. found that after 2 years, there was substantial improvement in the functional ability of the upper limbs and quality of life of patients with paresis (Wolf et al., 2008). In subacute stroke patients, CIMT was found to promote improvements in the function of the paralyzed hand (Treger et al., 2012b). In terms of frequency and training time, CIMT can improve motor function, amount of arm use, and upper limb self-efficacy after a stroke. Therefore, it seems to be an effective rehabilitation training method (Abdullahi, 2018). In terms of combination therapy, some scholars have explored the synergistic effect of CIMT and visual biofeedback training on subacute stroke patients. The results showed that CIMT plus visual biofeedback training did not significantly improve the upper limb function of patients with subacute stroke-induced hemiplegia. Larger sample sizes and different restriction durations are needed to elucidate this effect (Seok et al., 2016). In view of the absence of an active treatment control group in past studies on the effect of CIMT on upper limb function after stroke, Lin et al. designed a study that filled this gap; the results showed that CIMT greatly improved upper limb motor function, basic and extended functional abilities, and quality of life (Lin et al., 2009). In addition to investigating upper limb motor function improvements, scholars have studied the effect of CIMT on effective grasping and trunk control. Wu et al. (2012) found that CIMT improved grasping control and reversed compensatory trunk movements at the early stage of hand grasping movements, thereby generating additional benefits. da Silva et al. (2019) determined that moderate-to-high-intensity aerobic exercise improved the hand mobility of hemiplegic patients before mCIMT, providing valuable information for improving the fine skills of the upper limbs of stroke patients. Taub et al. (1993) independently observed the effect of CIMT on the upper limb function of chronic stroke patients and demonstrated that it was an effective strategy to restore substantial motor function. In addition, Taub et al. (2006) found that CIMT markedly improved the upper limb function of stroke patients in their daily lives (Wolf Motor Function Test), with the improvement effect lasting for 2 years. The transfer package of constraint-induced movement therapy is a method for enhancing both spontaneous use of a more affected arm after chronic stroke and its maximum motor capacity (Taub et al., 2013). Recently, CIMT has been applied in upper extremity rehabilitation for veterans with chronic or subchronic traumatic brain injury and achieved a satisfactory therapeutic effect (Morris et al., 2019).

Studies investigated the effect of CIMT on weight-bearing symmetry and balance in stroke patients in the sit-to-stand movement. The results indicated a greater load on the affected limb and a displacement of the center of pressure and center of mass (COM) toward the affected limb in the patients who had received CIMT. In the sit-to-stand movement, compliance disorders and asymmetric foot position may challenge the sagittal balance of stroke patients (Gray and Culham, 2014). The combined application of psychological training and mCIMT was shown to have a better rehabilitation effect (Kim et al., 2018). Compared with conventional rehabilitation therapy, mCIMT more effectively improved the upper limb function and occupational ability of stroke patients conventional rehabilitation therapy (Kim and Chang, 2018). Sheng et al. found that CIMT significantly improved upper limb motor function in stroke patients and confirmed, through functional magnetic resonance imaging (fMRI), that this change was related to changes in brain plasticity. When the healthy upper limb was restricted, motor function was not affected, and the excitatory area of the brain experienced transient changes (Sheng and Lin, 2009).

The above studies conducted in-depth research on the basic functions, duration of action, timing of intervention, and combined measures of CIMT and the effect of CIMT on activities of daily living, balance, occupational ability, and imaging, with no consensus results.

### Application of CIMT for the Lower Limbs

Emgs et al. investigated the effect of CIMT on lower limb functional activity and postural balance in stroke patients and found that a 2-week treadmill gait training regimen combined with home training effectively improved postural balance and functional activities in subacute stroke patients. However, the increase in load was not a differentiator between interventions (Emgs et al., 2017). In addition, Kallio et al. (2014) found that CIMT improved lower limb function and caused positive changes in balance and motor function in elderly chronic stroke patients. Zhu et al. (2016) evaluated the COM displacement and basic gait parameters of stroke patients and proposed that mCIMT intervention improved the COM displacement and improved hemiplegic gait parameters in stroke patients. Mark et al. (2013) found that CIMT was a safe and well-tolerated treatment for motor dysfunction of the lower extremities in patients with multiple sclerosis and significantly improved lower extremity function in 4 years.

### Other Applications

Self-regulation is an effective supplement to mCIMT. Liu et al. (2016) found that the combined application of self-regulation and mCIMT improved the functional recovery of stroke patients. Barzel et al. (2015) proposed that compared with conventional therapies, home-based CIMT more effectively improved the perceived use of the affected arm in daily activities in stroke patients but was not superior in improving motor function. In the context of addressing the patient perception that CIMT is boring, Choi et al. (2017) found that CIMT game training had a greater effect than regular game training on static balance control, load-bearing symmetry, and side-shifting weight (Choi et al., 2017). In terms of activities of daily living, Ju et al. found that improvements in hand function after mCIMT had a significant impact on feeding and dressing (Ju and Yoon, 2018). Nasb et al. (2019) showed that the therapeutic effect of botulinum-A toxin plus mCIMT on the recovery of motor function and activities of daily living in stroke patients was better than that of botulinum-A toxin plus intensified conventional therapy. CIMT has been shown to be effective in improving upper limb function in stroke patients with hemiplegia, but few studies have evaluated orthosis modifications. Kim et al. (2008) confirmed the efficacy of modified opposition restriction orthosis in chronic stroke patients with hemiplegia. By comparing the timing of CIMT intervention, Stock et al. found that in the long term, early intervention with CIMT was equally effective as delayed intervention with CIMT, especially in patients who reached an upper limit effect within 6 months after a stroke. However, the recovery of patients who underwent early CIMT intervention was faster than that of patients who underwent delayed CIMT intervention, a finding that has important clinical significance for patients in the acute phase (Stock et al., 2018). Similarly, adherence to CIMT is important. Stock et al. found that adherence was positively correlated with treatment progress and negatively correlated with age and that the use of gloves by female patients was low. These findings suggest that the parameters in CIMT regimens should be adjusted based on each patient in the early poststroke stage (Stock et al., 2015). Two studies focused on the long-term efficacy of CIMT, and both found that the effect of CIMT on improving limb function remained significant after 1 year (Wolf et al., 2006; Takebayashi et al., 2015). CIMT significantly improved aphasia patients’ speech abilities in daily life (Johnson et al., 2014).

Regarding imaging, some scholars proposed that CIMT changed the sensorimotor cortex activation (fMRI) and corticospinal conduction (transcranial magnetic stimulation) in chronic stroke patients. Moreover, the change was significantly correlated with clinical improvements in hand motor behavior (Könönen et al., 2012). Using fMRI, Zhao et al. found that the volume of the activated area increased after CIMT treatment. In addition to a reduction in the activated area around lesions, there were also newly activated areas, including in the auxiliary motor area, the premotor area and the ipsilateral sensorimotor area (Zhao et al., 2012). Treger et al. (2012a) found that the mean blood flow velocity in the injured middle cerebral artery significantly increased in stroke patients after the restriction of the unaffected hand. Some scholars used transcranial magnetic stimulation to assess changes in cortical excitability. The average Wolf Motor Function Test scores and MEPs after mCIMT were significantly higher than those at baseline. Compared with that in the control group, the ipsilateral silent period in the mCIMT group was significantly shorter (Yu et al., 2017).

## Limitations of CIMT

CIMT immediately after stroke and the immobilization of the ipsilateral forelimb caused the internal temperature of the brain tissue to increase by approximately 1°C, thereby aggravating brain injury in rats (DeBow et al., 2004). A small randomized controlled trial showed that only a small proportion of patients met the CIMT implementation criteria and that the use of upper limbs in functional tasks only slightly increased in the CIMT group (Baldwin et al., 2018). Brunner et al. (2012) found that 2-handed training had the same effect as mCIMT in improving arm motor function. For most poststroke patients in the subacute phase, gloves were unnecessary during intensive training with the affected arm. Thrane et al. (2015) reported that after a 6-month follow-up, CIMT resulted in no advantages regarding arm injury, function, or use in daily activities.

## Basic and Clinical Problems of CIMT and Prospects for CIMT

Based on the above studies, scholars have proposed a methodological solution to correct CIMT errors through decades of stringent systematic, multifaceted research (Morris et al., 2009). In basic research, the mechanisms by which CIMT plays a role are mainly angiogenesis and nerve regeneration. This topic is currently the main research focus of poststroke recovery. However, the evidence for CIMT effectiveness is not strong overall due to the lack of standardized manifestations of exercise therapy in cell experiments and the presence of many interfering factors and difficulties regarding the standardization of protocols in animal experiments. How can the movement amount and amplitude of the affected limb be made as consistent as possible after immobilization of the healthy limb? Does simple immobilization of the contralateral limb in animals have the same effect as the clinical restriction of the contralateral limb in humans? Answering these questions may require the joint efforts of scholars in statistics, animal behavior, and evidence-based medicine. Similarly, in clinical studies, the implementation of CIMT has certain requirements regarding muscle strength, muscle tone, and range of motion; therefore, CIMT can only be applied in certain conditions, limiting its wide clinical application. After restricting the physical activity of the healthy limb, the life of patients is greatly affected, with some patients exhibiting poor compliance, which may affect CIMT implementation and outcomes. After restricting the healthy limb, some patients experience depression because their activities of daily living are substantially affected, affecting their adherence to treatment. Is the cost–benefit ratio of CIMT appropriate? Does CIMT aggravate or induce poststroke depression? Does simply urging the patient to use the affected limb affect the coordination of the bilateral limbs and the ability to coordinate the activities of the bilateral limbs? Does CIMT weaken the driving effect of the contralateral limb on the affected side? Is CIMT partly contrary to the theory of mirror therapy? Last, the contralateral limb is currently restricted using different devices, including thick gloves and braces. Are these devices different regarding convenience or function? These problems have hindered the promotion and application of CIMT in clinical practice.

Rehabilitation medicine is an emerging discipline, and many rehabilitation training techniques are gradually improving through research and practice. Discovering problems in clinical practice and addressing and solving problems through basic research and clinical trials are the ways medical science progresses. CIMT is also gradually improving through these processes, and there may be stronger evidence in the future to support the application of CIMT in poststroke rehabilitation.

## Author Contributions

DW and WM conceived and supervised the study. DW, JX, YH, LD, and MY wrote the manuscript. ZY, DW, and MY made the manuscript revisions. All authors reviewed the results and approved the final version of the manuscript.

## Conflict of Interest

The authors declare that the research was conducted in the absence of any commercial or financial relationships that could be construed as a potential conflict of interest.

## Publisher’s Note

All claims expressed in this article are solely those of the authors and do not necessarily represent those of their affiliated organizations, or those of the publisher, the editors and the reviewers. Any product that may be evaluated in this article, or claim that may be made by its manufacturer, is not guaranteed or endorsed by the publisher.
